# Chronic Idiopathic Pancreatitis and Pancreaticolithiasis

**DOI:** 10.7759/cureus.106493

**Published:** 2026-04-05

**Authors:** Ruben Avanesian, Ripsime Movsesian, Edgar Didier Bandala Ruiz, Polina A Zholobova

**Affiliations:** 1 Department of General Surgery With Endoscopy, St. Petersburg State Pediatric Medical University, St. Petersburg, RUS; 2 Department of Planned Surgery, Mariinsky Hospital, St. Petersburg, RUS

**Keywords:** chronic pancreatitis, drainage of the pancreatic duct, lithiasic pancreatitis, pancreatic duct hypertension, puestow procedure

## Abstract

Chronic pancreatitis (CP) is an inflammatory syndrome that causes irreversible damage and loss of pancreatic function. A late complication is pancreatolithiasis, which exacerbates pain due to intraductal hypertension. In this work, we demonstrate that given the availability of different non-surgical treatment methods, if we are in the presence of dilated ducts, longitudinal pancreaticojejunostomy (Puestow procedure) is the best surgical drainage option. The clinical study is presented through the case of a 56-year-old man with idiopathic CP and recurrent lithiasis. The diagnosis was confirmed by magnetic resonance cholangiopancreatography. A lateral pancreaticojejunostomy was performed according to Puestow with Roux-Y anastomosis for ductal decompression. The intervention resolved ductal hypertension by allowing the drainage of liths. The patient was discharged after 10 days with improvement in liver profile and pancreatic enzymes, remaining asymptomatic during six months of follow-up. The Puestow procedure is a current and highly effective technique for refractory lithiasis CP. It is feasible in patients with stones larger than 2 mm, allowing complete functional recovery and preventing secondary complications.

## Introduction

Chronic pancreatitis (CP) encompasses a set of clinical manifestations secondary to an uninterrupted inflammatory process of the pancreas. The American Pancreas Association (APA) defines it as: "an inflammatory syndrome and chronic progressive scarring of the pancreas, with irreversible damage and loss of exocrine and endocrine function" [1,2]. There are several etiologies attributed to the development of chronic pancreatitis. Alcohol consumption is attributed to a high number of reported cases. Other causes are included in the acronym TIGAR-O (Toxic/Metabolic, Idiopathic, Genetic, Autoimmune, Recurrent Severe Acute Pancreatitis, Obstructive). Chronic idiopathic pancreatitis (CIP) is diagnosed in the absence of obvious precipitating factors [3,4]. Among the late complications of chronic pancreatitis, we have pancreatolithiasis, which exacerbates chronic abdominal pain due to intraductal hypertension and parenchymal ischemia [5,6]. The Puestow procedure was first described in 1958 by Partington and Rochelle. It is a drainage procedure that performs a side-to-side longitudinal pancreaticojejunostomy. This type of surgical procedure is the best option when there is dilation of the main pancreatic duct (minimum diameter of 5 mm), in the absence of an inflammatory mass in the head of the pancreas or obstruction of the bile duct [3,6]. In this clinical case, the complexity of chronic pancreatitis is highlighted. Understanding the pathophysiology and complications is critical, as is the importance of timely diagnosis and treatment to improve patients' quality of life.

## Case presentation

A 56-year-old man with a history of grade 2 hypertension, atherosclerosis, chronic gastritis, and a previous diagnosis of chronic idiopathic pancreatitis and intraductal pancreatic hypertension since April 2023. The surgical/interventional history included a puncture and external drainage of the pancreatic duct, failed attempts at endoscopic dilations with bougienage and balloons, and the placement of a percutaneous transgastric drainage due to persistent pain.

**Figure 1:**
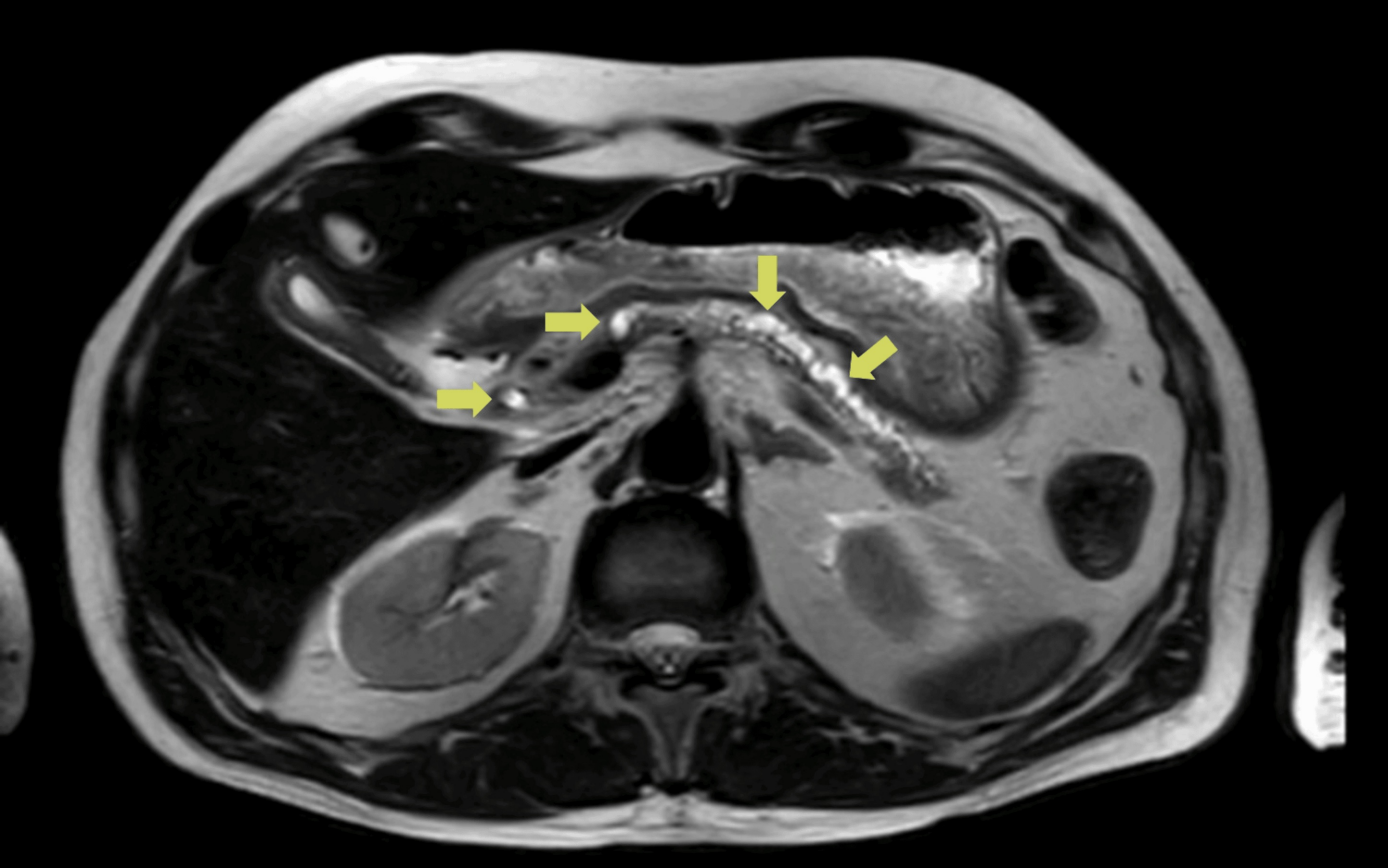
Preoperative MRI Multiple liths are observed along the Wirsung duct (marked with green arrows).

The patient was admitted with a three-day history characterized by sudden epigastric pain radiating in a belt to the posterior thorax (visual analog scale (VAS) 9/10) and vomiting food. On examination, he presented a pained expression and a painful abdomen on palpation in the epigastrium and mesogastrium, although soft and without palpable masses. Laboratory studies revealed an elevation of pancreatic enzymes and a cholestasis profile (Table 1). Abdominal ultrasound showed pancreatic ductal ectasia and multiple hyperechogenic images in the head and body of the pancreas; on the other hand, magnetic resonance cholangiopancreatography (MRCP) confirmed chronic atrophic pancreatitis and pancreatolithiasis (liths up to 0.3 cm in the pancreatic duct that we can see in Figure 1 marked with green arrows) with dilation of the extrahepatic bile ducts.

**Table 1:** Laboratory studies mg/dL = milligrams per deciliter; IU/L = international units per liter; g/dL = grams per deciliter

Laboratories		
Liver Function Profile		
	Result	Reference Ranges
Total bilirubin	4.7 mg/dL	<1.2 mg/dL
Direct bilirubin	3.6 mg/dL	0.09 - 0.3 mg/dL
Indirect bilirubin	1.1 mg/dL	0.01 - 0.9 mg/dL
Aspartate aminotransferase (AST)	19 IU/L	<40 IU/L
Alanine aminotransferase (ALT)	29 IU/L	<41 IU/L
GGT glutamyl transpeptidase range	83 IU/L	9 - 75 IU/L
Total protein	6.9 g/dL	6.3 - 8.1 g/dL
Albumin	4.7 g/dL	3.9 - 5.1 g/dL
Alkaline phosphatase (AF)	286 IU/L	17 - 142 IU/L
Pancreatic Enzymes		
	Result	Reference Ranges
Amylase	518 IU/L	35 - 127 IU/L
Lipase	437 IU/L	18 - 140 IU/L

**Figure 2:**
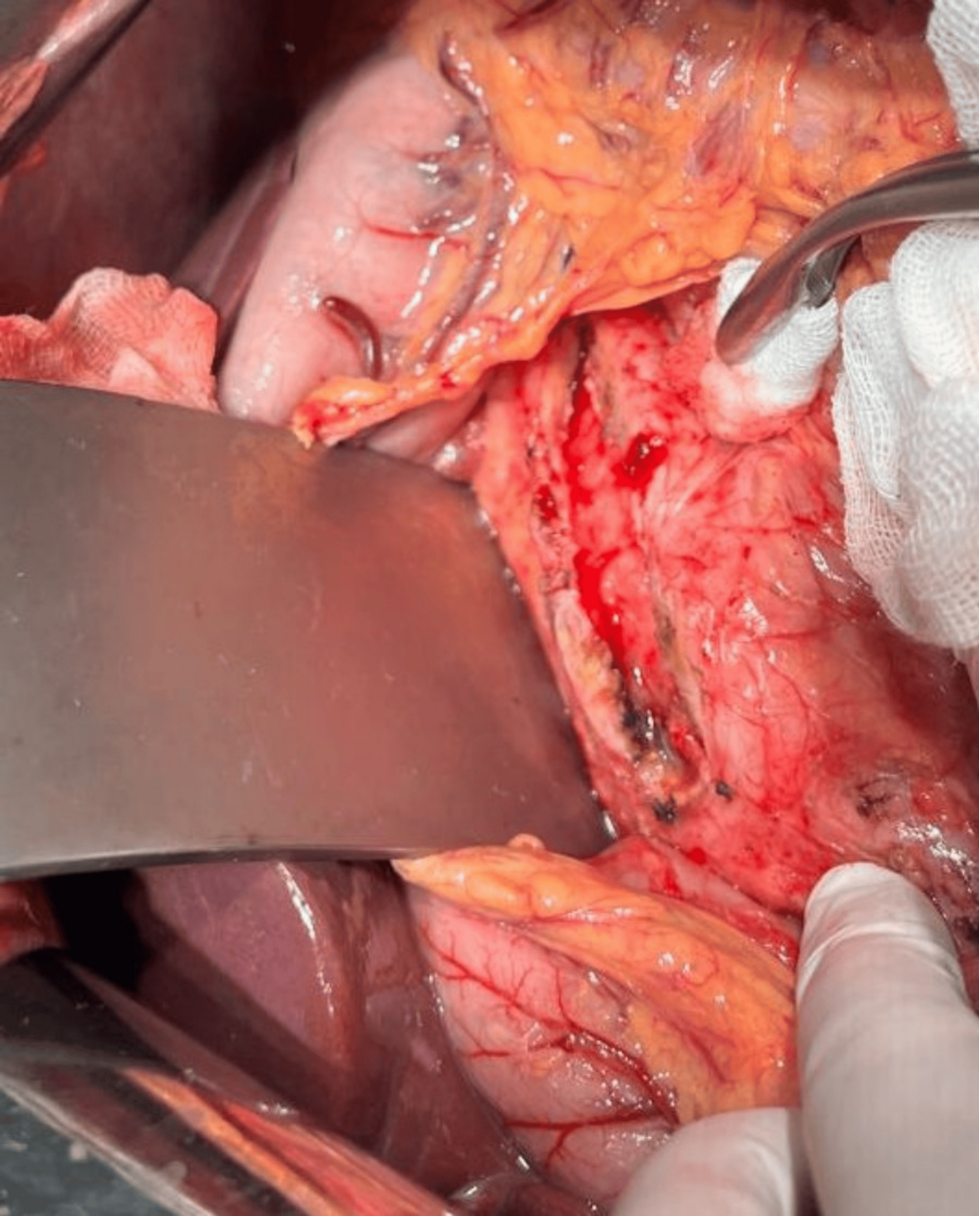
Side-to-side incision along the duct of Wirsung

**Figure 3:**
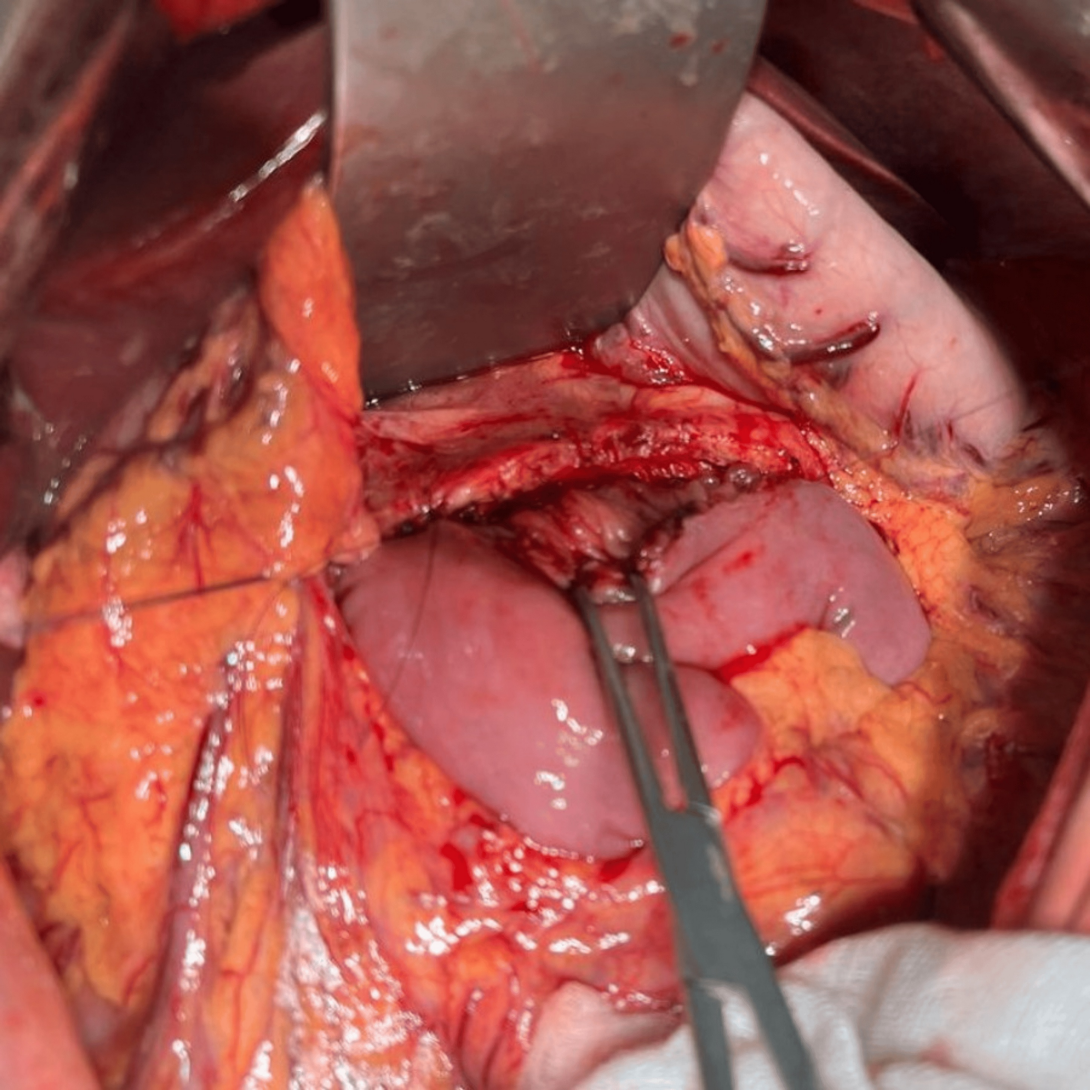
Side-to-side anastomosis between the pancreas and jejunum

**Figure 4:**
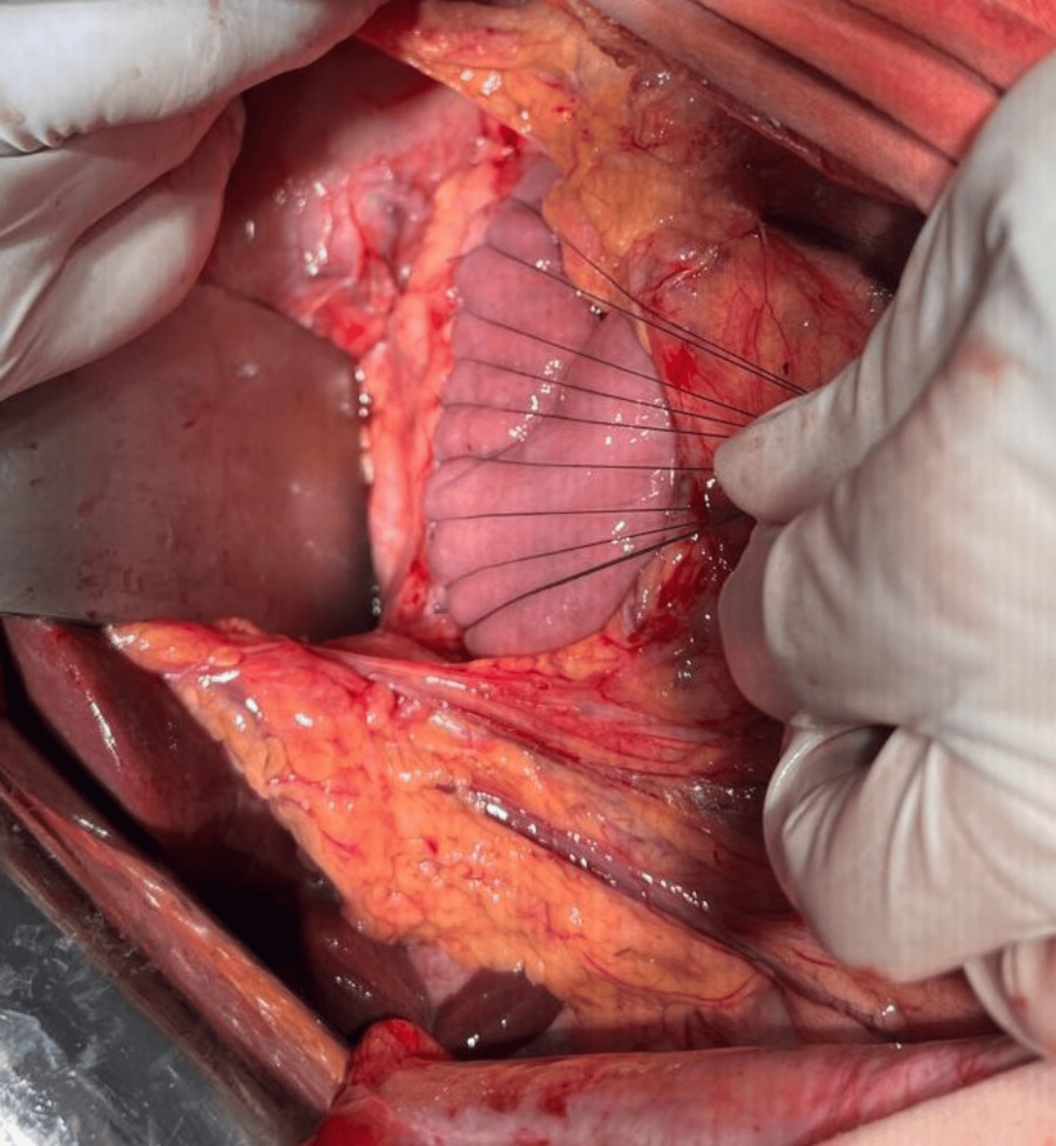
Side-to-side anastomosis between the pancreas and jejunum

Given the recurrence of symptoms and imaging evidence of ductal hypertension due to lithiasis, a lateral pancreaticojejunostomy was performed on March 1, 2025, according to Puestow with Roux-en-Y anastomosis (Figures 2-4). This procedure focused on longitudinal decompression of the main pancreatic duct to facilitate drainage of pancreatic flow and liths.

The postoperative period passed without significant alterations in renal function, electrolytes, or coagulation tests. After observing a substantial improvement in the liver profile and pancreatic enzymes, the patient was discharged 10 days after the intervention. In the outpatient follow-up for six months, the patient remained asymptomatic, with no new episodes of pancreatitis.

## Discussion

Lara-Orozco U et al. characterize chronic pancreatitis as a pathological fibroinflammatory syndrome of the pancreas in individuals with genetic, environmental, or other risk factors who develop persistent pathological responses to parenchymal lesions or stress. An overall incidence of 4 to 14 per 100,000 people per year is estimated. Alcohol consumption (> 5 drinks/day) is the most important risk factor for its development, causing 70% of cases in adults [2]. The most frequent clinical manifestations are abdominal pain and stereorhea. When there is approximately 90% pancreatic dysfunction, the tendency to manifest signs of exocrine dysfunction (steatorrhea, malabsorption, and deficiencies of fat-soluble vitamins) increases similarly, due to increased fibrosis. Patients may also develop endocrine dysfunction, developing pancreatogenic diabetes. As a result of pain-related fat malabsorption and anorexia, weight loss and malnutrition are frequently found in patients with chronic pancreatitis [2].

CIP, also defined as chronic pancreatitis in the absence of obvious precipitating factors, can be classified according to Aguado Valerio JA et al. as early-onset idiopathic chronic pancreatitis (EOICP) in patients under 35 years of age and late-onset idiopathic chronic pancreatitis (LOICP) in patients over 35 years of age according to a bimodal presentation phenomenon associated with different predominant features of clinical symptoms in each age group; the difference is that in EOICP, high frequency and severity of pain is observed with slower-onset exocrine and endocrine dysfunction, and in LOICP, there is no pain in up to 50% of cases and develops with varying degrees of pancreatic dysfunction during the course of the disease [4].

Endoscopic ultrasound (EUS) is considered the most sensitive method for diagnosing chronic pancreatitis. Eleven criteria describing parenchymal and ductal features are used to classify chronic pancreatitis. The Rosemont system provides a regulatory framework for the endoscopic identification of chronic pancreatitis, employing specific endoscopic ultrasonography (EUS) metrics to stratify patients based on their likelihood of developing this pathology. It classifies ultrasound findings into categories of major and minor significance, distinguishing between abnormalities of the pancreatic parenchyma and those of the ductal system. In the evaluation of the parenchyma, the signs of greatest clinical relevance (Major A and B) include the presence of hyperechoic foci casting acoustic shadowing and honeycomb-like lobulation; conversely, features such as cysts, hyperechoic bands, and simple lobulation are classified as minor criteria. With regard to ductal features, the detection of stones in the duct is established as the primary major finding, while dilation of the main pancreatic duct or its lateral branches, along with hyperechoic margins and irregular contours, serves as a minor indicator of the disease [7].

The final diagnostic interpretation is based on a quantitative and qualitative combination of these findings to determine the likelihood of the condition. A case is defined as consistent with chronic pancreatitis when two major A criteria are present, a combination of one major A and one major B, or a major A criterion supported by more than three minor signs. The study is considered suggestive of the disease if a major A criterion is identified with fewer than three minor signs, a major B criterion associated with more than three minor signs, or simply the presence of five or more minor features of any type. Finally, results are classified as indeterminate in the presence of three to four minor signs without major criteria, or an isolated major B sign; the pancreas is reported as normal only when there are fewer than two minor findings and a total absence of major criteria [7].

Despite its importance in diagnostic standardization, the ability of this model to predict clinical evolution or the success of therapeutic interventions remains, to date, a question in medical practice [3,7].

As a consequence of the prolonged evolution of chronic pancreatitis, it is common to observe the development of pancreatolithiasis as a late sequelae. This pathology involves the formation of stones that can be located from the pancreatic parenchyma itself and the lateral ductal branches to the pancreatic ducts of Wirsung and Santorini [3]. Brebu et al. argue that the reverberant inflammatory process, followed by fibrotic degeneration of the pancreatic parenchyma and stasis of the pancreatic fluid at the ductal level, are factors that contribute to the phenomenon of calcium precipitation, since there is a high concentration of calcium in the pancreatic juice, which is kept within physiological limits thanks to the intervention of bicarbonate (HCO3), citrate and protein from pancreatic stones. In chronic pancreatitis, regulatory mechanisms are compromised by the destruction of the parenchyma, a phenomenon that results in the appearance of pancreaticolithiasis. When these lithiasic formations lodge in the duct of Wirsung, they block the normal transit of secretions, triggering ductal hypertension. This obstruction not only prevents flow through the pancreatic duct but also induces ischemic degeneration in the organ, which intensifies the clinical picture and persistent pain in the patient [3].

According to the available scientific evidence, the diagnosis of pancreatolithiasis is based on the use of advanced imaging techniques; specifically, endoscopic ultrasound and magnetic resonance cholangiopancreatography stand out for their remarkable detection capacity and high sensitivity [3,7].

For the treatment of pancreaticolithiasis, Lara-Orozco et al. state that surgical treatment is more effective and long-lasting than endoscopic treatment [2]. On the other hand, Brito-Carmona et al. mention the groupings of current surgical options that consist of drainage, resection, resection with prolonged drainage, or pancreatic denervation procedures; the choice of procedure depends on the morphological changes of the pancreas (dilation of the Wirsung's duct, stenosis, lithiasis) [5].

The lateral pancreaticojejunostomy, or Puestow procedure, has been the milestone in the surgical treatment of chronic fibrocalcific pancreatitis for more than half a century, thanks to its low morbidity and mortality and success in relieving the pain associated with chronic pancreatitis. Patients with a pancreatic duct diameter greater than 5 mm are candidates for this surgical method. The surgical technique useful today, which Puestow and Gillesby described in 1958, is developed as follows: they resected a portion of the tail of the pancreas that was sent for pathological examination. The pancreatic duct was opened as far to the right as possible. The Roux-en-Y jejunal loop was introduced through the transverse mesocolon. Anastomosis was performed with cotton sutures interrupted in two layers, and the anastomosis was drained with a split Penrose drain [6].

Today, authors such as McCarron et al. mention that for a procedure like Puestow's, robotic surgery also transforms a traditionally open surgical procedure into a minimally invasive procedure that maintains, or even improves, the precision of reconstruction thanks to the vision technology of the instruments. In a lateral pancreatojejunostomy, which requires a long longitudinal anastomosis between the pancreatic duct and jejunum, the improved dexterity provided by articulated instruments is a significant advantage over conventional surgery [8].

Although endoscopy is preferred as initial treatment due to its lower invasiveness and fewer complications, it has disadvantages that include a lower rate of long-term pain relief and a greater need for further interventions compared to surgery, especially in patients with multiple large calculi, complex strictures, or extensive disease. Surgery should be reserved for cases in which endoscopy and extracorporeal shock wave lithotripsy (ESWL) fail or in patients with unfavorable anatomy for an endoscopic approach [9,10].

This case underscores the importance of a timely transition from endoscopic to surgical management in chronic pancreatitis. The medical literature suggests that, in patients with a dilated main pancreatic duct, drainage surgery, such as the Puestow procedure, offers longer-lasting pain relief compared to repeated endoscopic interventions. The presence of multiple pancreatolithiasis hindered the success of conservative management, making surgical decompression the most effective option for resolving pancreatic hypertension.

## Conclusions

Lateral pancreaticojejunostomy continues to be a current and highly effective surgical technique for the management of refractory chronic lithiasic pancreatitis. In this patient, the intervention not only resolved the mechanical obstruction but also allowed for full functional recovery and a significant improvement in short-term quality of life.

In conclusion, the treatment of patients affected by pancreatolithiasis currently lacks a universally accepted clinical guideline, given the lack of a global consensus on its management. However, the evidence from this study supports that longitudinal pancreaticojejunostomy, based on the Puestow method, is a highly effective and executable surgical alternative. This technique is especially recommended in cases of recurrent lithiasis where the stones exceed 2 mm in size. Its implementation allows not only the mitigation of symptoms and a substantial improvement in the patient's quality of life but also works as an essential measure to prevent additional complications derived from the progression of chronic pancreatitis.
